# Supplementary material for: Iterative improvement in the automatic modular design of robot swarms
Source: PeerJ Comput Sci. 2020 Dec 7;6:e322. doi: 10.7717/peerj-cs.322 (PMC7924708; doi:10.7717/peerj-cs.322)
Supplement: Supplemental Information 3 [file peerj-cs-06-322-s003.zip › argos3/doc/api/standalone/a00633_source.html]

ARGoS: plugins/simulator/visualizations/qt-opengl/models/qtopengl\_light.cpp Source File


- Main Page
- Related Pages
- Namespaces
- Classes
- Files

- File List
- File Members

# plugins/simulator/visualizations/qt-opengl/models/qtopengl\_light.cpp

Go to the documentation of this file.

```
00001 
00007 #include "qtopengl_light.h"
00008 #include <argos3/core/utility/math/vector3.h>
00009 #include <argos3/plugins/simulator/entities/light_entity.h>
00010 #include <argos3/plugins/simulator/visualizations/qt-opengl/qtopengl_widget.h>
00011 
00012 namespace argos {
00013 
00014    /****************************************/
00015    /****************************************/
00016 
00017    CQTOpenGLLight::CQTOpenGLLight() :
00018       m_unVertexes(20),
00019       m_fRadius(0.1f) {
00020       /* Reserve a display list */
00021       m_unList = glGenLists(1);
00022 
00023       /* Start the display list */
00024       glNewList(m_unList, GL_COMPILE);
00025 
00026       /* Let's start the actual shape, a sphere */
00027       CVector3 cNormal, cPoint;
00028       CRadians cSlice(CRadians::TWO_PI / m_unVertexes);
00029 
00030       glBegin(GL_TRIANGLE_STRIP);
00031       for(CRadians cInclination; cInclination <= CRadians::PI; cInclination += cSlice) {
00032          for(CRadians cAzimuth; cAzimuth <= CRadians::TWO_PI; cAzimuth += cSlice) {
00033 
00034             cNormal.FromSphericalCoords(1.0f, cInclination, cAzimuth);
00035             cPoint = m_fRadius * cNormal;
00036             glNormal3f(cNormal.GetX(), cNormal.GetY(), cNormal.GetZ());
00037             glVertex3f(cPoint.GetX(), cPoint.GetY(), cPoint.GetZ());
00038 
00039             cNormal.FromSphericalCoords(1.0f, cInclination + cSlice, cAzimuth);
00040             cPoint = m_fRadius * cNormal;
00041             glNormal3f(cNormal.GetX(), cNormal.GetY(), cNormal.GetZ());
00042             glVertex3f(cPoint.GetX(), cPoint.GetY(), cPoint.GetZ());
00043 
00044             cNormal.FromSphericalCoords(1.0f, cInclination, cAzimuth + cSlice);
00045             cPoint = m_fRadius * cNormal;
00046             glNormal3f(cNormal.GetX(), cNormal.GetY(), cNormal.GetZ());
00047             glVertex3f(cPoint.GetX(), cPoint.GetY(), cPoint.GetZ());
00048 
00049             cNormal.FromSphericalCoords(1.0f, cInclination + cSlice, cAzimuth + cSlice);
00050             cPoint = m_fRadius * cNormal;
00051             glNormal3f(cNormal.GetX(), cNormal.GetY(), cNormal.GetZ());
00052             glVertex3f(cPoint.GetX(), cPoint.GetY(), cPoint.GetZ());
00053 
00054          }
00055       }
00056       glEnd();
00057 
00058       /* End of the display list */
00059       glEndList();
00060    }
00061 
00062    /****************************************/
00063    /****************************************/
00064 
00065    CQTOpenGLLight::~CQTOpenGLLight() {
00066       glDeleteLists(m_unList, 1);
00067    }
00068 
00069    /****************************************/
00070    /****************************************/
00071 
00072    void CQTOpenGLLight::Draw(CLightEntity& c_entity) {
00073       /* Set the material */
00074       const CColor& cColor = c_entity.GetColor();
00075       const GLfloat pfColor[]     = { cColor.GetRed()   / 255.0f,
00076                                       cColor.GetGreen() / 255.0f,
00077                                       cColor.GetBlue()  / 255.0f,
00078                                       1.0f };
00079       const GLfloat pfSpecular[]  = { 0.0f,  0.0f, 0.0f, 1.0f };
00080       const GLfloat pfShininess[] = { 100.0f                  };
00081       const GLfloat pfEmission[]  = { 0.0f,  0.0f, 0.0f, 1.0f };
00082       glMaterialfv(GL_FRONT_AND_BACK, GL_AMBIENT_AND_DIFFUSE, pfColor);
00083       glMaterialfv(GL_FRONT_AND_BACK, GL_SPECULAR, pfSpecular);
00084       glMaterialfv(GL_FRONT_AND_BACK, GL_SHININESS, pfShininess);
00085       glMaterialfv(GL_FRONT_AND_BACK, GL_EMISSION, pfEmission);
00086 
00087       glCallList(m_unList);
00088    }
00089 
00090    /****************************************/
00091    /****************************************/
00092 
00093    class CQTOpenGLOperationDrawLightNormal : public CQTOpenGLOperationDrawNormal {
00094    public:
00095       void ApplyTo(CQTOpenGLWidget& c_visualization,
00096                    CLightEntity& c_entity) {
00097          static CQTOpenGLLight m_cModel;
00098          c_visualization.DrawEntity(c_entity);
00099          m_cModel.Draw(c_entity);
00100       }
00101    };
00102 
00103    class CQTOpenGLOperationDrawLightSelected : public CQTOpenGLOperationDrawSelected {
00104    public:
00105       void ApplyTo(CQTOpenGLWidget& c_visualization,
00106                    CLightEntity& c_entity) {
00107          static CQTOpenGLLight m_cModel;
00108          glPolygonMode(GL_FRONT_AND_BACK, GL_LINE);
00109          c_visualization.DrawEntity(c_entity);
00110          glScalef(1.1, 1.1, 1.1);
00111          m_cModel.Draw(c_entity);
00112          glPolygonMode(GL_FRONT_AND_BACK, GL_FILL);
00113       }
00114    };
00115 
00116    REGISTER_QTOPENGL_ENTITY_OPERATION(CQTOpenGLOperationDrawNormal, CQTOpenGLOperationDrawLightNormal, CLightEntity);
00117 
00118    REGISTER_QTOPENGL_ENTITY_OPERATION(CQTOpenGLOperationDrawSelected, CQTOpenGLOperationDrawLightSelected, CLightEntity);
00119 
00120    /****************************************/
00121    /****************************************/
00122 
00123 }
```

---

Generated on 10 Jul 2018 for ARGoS by 
 1.6.1
